# State Control in Recruiting Probationers

**Published:** 1920-10-09

**Authors:** 


### STATE CONTROL IN RECRUITING PROBATIONERS.

(By a Matron and College Member.)

In every training-school throughout the country there is a terrible shortage of probationers, apparently candidates desirous of entering the nursing services are rapidly becoming non-existent. Week after week, through the medium of the nursing Press, matrons advertise their vacancies, seemingly with little result. Committees face to face with changed economic conditions are compelled to raise the salaries of all ranks and to shorten the hours of the working-week. Hospital competes with hospital in its endeavour to lure within the fold women who are willing to undergo the requisite training which will enable them to become qualified and certificated nurses.

Obviously this policy of competition, even if it were successful—which apparently it is not—cannot continue indefinitely. Even now the tension is at breaking-point; the entire system may at any moment collapse. It is easy to say the machinery is obsolete and it must be scrapped; but, before scrapping a system, it is advisable to evolve a scheme by which it may be replaced; and hospital committees apparently have no alternative to offer. The matter is daily becoming more urgent; the nursing of the sick is vital to the State, and the State cannot afford to remain indifferent to the exigency of the moment; it must and will act.

### How the State might Help.

Shall we see in the near future, under the ægis of State control, the co-ordination of the curriculum of the various training-schools and the evolution of a civilian nursing service, the professional and economic status of which is guaranteed by the State? Surely it is the only feasible solution of the present-day problem, and would tend to increase rather than to destroy that elusive gift of *esprit de corps* which binds together the various sections of the nursing services. The conditions of service could be advertised in the daily Press, and would-be candidates would receive on application to headquarters, a syllabus containing information relating not only to the entrance examination, but also to the conditions of service during training; a syllabus of the instruction to be imparted, theoretically and practically; the hours they would be on duty, the salary they would receive, and the posts for which they would be eligible on the completion of their training. Candidates would be invited to submit in the order of their preference a list of the hospitals in which they desired to train, and, according to their place on the pass-list, they would be allocated to the training-school they preferred, provided they were successful in passing the medical and selection committees. The selection committee would be composed of representatives selected by the various training-schools, and they would be empowered, if the health of a nurse in training would not stand the strain of a large training-school, to grant her facilities to enable her to finish her training in a smaller hospital approved by headquarters, and so obviate the injustice of a woman after eighteen months' or two years' service being turned adrift half-trained owing to physical disability occasioned by her work.

A scheme as the above would assuredly benefit all classes. Matrons and hospital committees would receive as probationers women of recognised educational status, while the nurses themselves would have their interests safeguarded by State control. At the conclusion of their curriculum they would be required to pass a public examination which would further hall-mark them as civil servants of no mean standing, and successfully checkmate all attempts at exploitation.

